# Will whole-genome sequencing become the first-line genetic analysis for male infertility in the near future?

**DOI:** 10.1186/s12610-021-00138-4

**Published:** 2021-08-19

**Authors:** Farah Ghieh, Anne-Laure Barbotin, Clara Leroy, François Marcelli, Nelly Swierkowsky-Blanchard, Valérie Serazin, Béatrice Mandon-Pepin, François Vialard

**Affiliations:** 1 UVSQ, INRAE, BREED, Université Paris-Saclay, F-78350 Jouy-en-Josas, France; 2 Ecole Nationale Vétérinaire d’Alfort, BREED, F-94700 Maisons-Alfort, France; 3 Institut de Biologie de la Reproduction-Spermiologie-CECOS, Hôpital Jeanne de Flandre, Centre Hospitalier et Universitaire, F -59000 Lille, France; 4 Département de Gynécologie-Obstétrique, CHI de Poissy-St Germain en Laye, F-78300 Poissy, France; 5 Département de Génétique, Laboratoire de Biologie Médicale, CHI de Poissy-St Germain en Laye, F-78300 Poissy, France

**Keywords:** Male infertility, Whole-genome sequencing, Azoospermia, Macrozoospermia, Globozoospermia, Multiple morphological abnormalities of the flagella, Infertilité masculine, Séquençage complet du génome (WES), Azoospermie, Macrozoospermie, Globozoospermie, Anomalies morphologiques multiples du flagelle

## Abstract

Whereas the initially strategy for the genetic analysis of male infertility was based on a candidate gene approach, the development of next-generation sequencing technologies (such as whole-exome sequencing (WES)) provides an opportunity to analyze many genes in a single procedure. In order to recommend WES or whole-genome sequencing (WGS) after genetic counselling, an objective evaluation of the current genetic screening strategy for male infertility is required, even if, at present, we have to take into consideration the complexity of such a procedure, not discussed in this commentary.

## Résumé

Alors que la stratégie actuelle d'analyse de génétique moléculaire de l'infertilité masculine est basée sur une approche dite "gène candidat", le développement des technologies de séquençage de nouvelle génération, comme le séquençage complet de l'exome (WES), offre la possibilité d'analyser de nombreux gènes en une seule technique.

Afin de recommander le WES ou le séquençage complet du génome, après un conseil génétique, une évaluation objective des différentes stratégies de dépistage génétique est nécessaire, tout en prenant en considération que la complexité d'une utilisation des nouvelles technologies n'est pas abordé dans ce commentaire.

About 50% of cases of infertility are of male origin [1]. The infertility is due to quantitative and/or qualitative abnormalities in spermatogenesis, which affect the sperm count, motility and/or morphology (resulting in oligo/azoospermia, asthenozoospermia, and teratozoospermia, respectively). During the 1970s, the karyotyping of infertile men led to the observation of many types of chromosome rearrangement [2]. Several anomalies involve the sex chromosomes or feature autosomal Robertsonian translocations. The lower the sperm count, the higher the frequency of chromosomal anomalies; this is mainly due to Klinefelter syndrome, which is observed in 15% of men with azoospermia. With the emergence of new technologies and new data processing methods, genetic screening now has a broader scope. Ever since karyotyping revealed that Y chromosome rearrangements involving the long arm are associated with infertility, researchers had expected to find an azoospermia factor (AZF). Indeed, three regions (AZFa, AZFb, and AZFc) were subsequently identified [3]. According to the European Academy of Andrology and the European Molecular Genetics Quality Network guidelines, men with a sperm count below 5 million per ml should be screened for AZF defects [4]. Given that almost all men with cystic fibrosis (CF) have a congenital bilateral absence of the vas deferens (CBAVD), it has further been hypothesized that isolated CBAVD (OMIM#277180) is a distinct genetic entity associated with an elevated frequency of CF gene mutations [5]. Hence, CBAVD is now referred to as a *CFTR*-related disease.

With the emergence of whole-genome molecular analyses, a variety of different syndromes and single nucleotide variants (SNVs) associated with male infertility have been described. These include SNVs in the gene coding for aurora kinase C (*AURKC*) for most cases of macrozoospermia [6], and copy number variations (CNVs) or SNVs in the dpy-19-like 2 gene (*DPY19L2*) for most cases of globozoospermia [7].

Although the initial screening strategy was based on a candidate gene approach, the development of next-generation sequencing (NGS) technologies (such as whole-exome sequencing (WES)) provides an opportunity to analyze many genes in a single procedure. In the near future, whole genome sequencing (WGS) will enable the simultaneous diagnosis of chromosome rearrangements, CNVs, and SNVs. Since the candidate gene screening approach is time-consuming, it must be restricted to a single gene strategy - particularly for genes with fewer exons and/or variant hot spots. In other situations, one or more of three NGS techniques should be favored: targeted sequencing (TS), WES and WGS, as the technical cost issue tends to fall over time. It should be noted that the longer the sequence, the highest the number of genetic variants, and consequently the longer and more expensive the data analysis and interpretation.

However, an objective evaluation of the today’s genetic screening strategy for male infertility is necessary in order to recommend (or not) WGS - even though it is still a long, expensive process. This evaluation can be chosen as a function of the sperm phenotype. Furthermore, the implementation of both WGS and WES should always be preceded by genetic counselling. The purpose of the analysis must be explained clearly to the patient because WES or WGS can potentially identify gene defects unrelated to infertility (e.g. predispositions to cancer or early-onset neurodegenerative diseases).

Finally, we must keep in mind that the complexity of patient consent, the requirement for pre- and post-test genetic counseling, the complexity of data interpretation, the complexity of patient feedback, and the family implications are likely to be a limitation to implementation in the near future. All of these points will not be addressed in the commentary although they will be the subject of much discussion in the future.

## Quantitative defects

### Azoospermia

Azoospermia is defined as the total absence of spermatozoa in the ejaculate in two successive semen examinations. It accounts for around 10% of cases of male infertility, and affects about 1% of the men in the general population [8–10]. The condition can be classified as non-obstructive azoospermia (NOA, associated with spermatogenesis failure, and accounting for 60% of cases) or obstructive azoospermia (OA, characterized by normal spermatogenesis and an obstruction in the seminal tract, and accounting for the remaining 40%) [11, 12]. In around 95% of cases of azoospermia, the combination of testicular sperm extraction (TESE) with in vitro fertilization (IVF) and intracytoplasmic sperm injection (ICSI) gives the patient an opportunity to become a father [12].

With the exception of men with congenital bilateral (CBAVD) or unilateral absence of the vas deferens (OMIM #277180), men with OA do not currently undergo genetic screening because the condition is generally due to chronic infection and/or inflammation of the ejaculatory tract. In contrast, full sequencing of the *CFTR* gene is recommended for a man for CAVD and his partner, in order to evaluate the risk of CF in the offspring [13]. Eighty percent of men with CBAVD carry one or two *CFTR* mutations [13], and it is impossible to consider TESE before genetic testing and counseling. Furthermore, a quarter of the men without a *CFTR* gene mutation may have a defect in the *ADGRG2* gene associated with X-linked CBAVD (OMIM #300985) [14]. Although various candidate genes (such as *PANK2* and *SLC9A3*) have been suggested in the literature [15], none has been considered for CBAVD diagnosis. Therefore, in this configuration, only TS of *CFTR* and *ADGRG2* would be necessary.

While the clinical validity of *ADGRG2* screening is restricted to the etiologic diagnosis of OA (with no associated change in patient care), *CFTR* screening is clearly associated with great benefit in routine clinical practice. If the partner carries a CF mutation, preimplantation or prenatal diagnosis should be considered before an IVF procedure. WES or WGS should be used for research purposes and not in clinical practice, since there is no patient benefit.

For men with NOA, the sperm retrieval rate in TESE is around 40 to 50%. After the exclusion of acquired diseases such as cryptorchidy or varicocele, genetic screening can be suggested. Many genetic defects are associated with this condition, and TESE may be contraindicated by the genetic testing results: a 46,XX karyotype (usually 46,X,der(X)t(X;Y)(p22.3;p11.2) results from an unbalanced de novo X-Y translocation and the translocation of the sex-determining region of the Y chromosome to the X chromosome), AZFa and/or AZFb microdeletions, leading respectively to Sertoli-cell-only syndrome and sperm maturation arrest [16]. Other chromosome abnormalities (such as Klinefelter syndrome) do not contraindicate TESE, although genetic counselling is required to evaluate the risk of an unbalanced karyotype in the offspring; this is mainly applied to reciprocal or Robertsonian translocations and inversions. Hence, karyotyping and Y chromosome microdeletion screening [2, 17] are currently considered as first-line analyses.

However, this strategy gives a diagnosis in only 15% of cases, and the emergence of NGS will probably increase this rate. WES has led to a great increase in the number of different gene defects reported [15] but none currently have clinical implications. Recurrent abnormalities in genes coding for synaptonemal complex central element protein 1 (*SYCE1*), meiotic double-stranded break formation protein 1 (*MEI1*), stromal antigen 3 (*STAG3*), and testis expressed-11 (*TEX11*), *TEX14*, and *TEX15* have now been described [18, 19] (mainly in consanguineous families, except for *TEX11*).

TS has also been evaluated [18]; although many variants have been reported, the great majority are considered to be variants of unknown significance (VUS) or benign variants with no clinical value. Most of them are too frequent in the overall population (up to 0.1%) and/or only are predicted to have a small effect on the encoded protein. A focus on genes only involved on spermatogenesis helps to avoid the fortuitous discovery of gene defects associated with other pathologies (such as cancer), consider only actionable variants and avoid unwarranted TESE, and consume less time. However, WES could be considered in the near future as a second-line genetic analysis (after karyotyping and Y-chromosome microdeletion screening, especially for consanguineous men), even though the discovery of SNVs does not modify the clinical practice and avoid TESE (with the exception of recurrently reported *TEX11* defects), (i) genetic analysis software that reports only class 4 and 5 variants (according to the American College of Medical Genetics (ACMG) guidelines [20] or VUS for spermatogenesis-specific genes is likely to be developed in the near future, and (ii) a large number of genes are involved in spermatogenesis [21]. The identification of gene defects will facilitate discussion with the patient about the risk/benefit balance for TESE. While more time will probably be needed to contraindicate TESE following the identification of a pathologic variant, this approach will clearly improve clinical practice - especially after an unsuccessful first attempt in which a deleterious SNV is associated with a homogeneous histological profile (such as maturation arrest or Sertoli-cell-only syndrome).

In fact, WGS could replace all these techniques and become the first-line analysis because of (i) its ongoing implementation, (ii) the development of software that facilitates the interpretation of genetic test results and the identification of all genetic variants (SNVs, CNVs, and chromosome structure rearrangements), and (iii) lower costs (correlated with deeper sequencing).

### Oligozoospermia

Given (i) the lower frequency of chromosome rearrangements and Y chromosome microdeletions, (ii) the absence of known gene defects associated with this condition, and (iii) the absence of contraindication for medically assisted reproduction, karyotyping alone should be suggested as a guide to the etiology of oligozoospermia. Subsequent genetic counseling can evaluate the risk for the offspring (see the previous paragraph) as a function of the type of chromosomal segregation during meiosis. WES and WGS appeared to be restricted to research programs and are not used in routine clinical practice.

## Teratozoospermia

Here, we only considered homogeneous teratozoospermia, i.e., conditions in which more than 99% of the spermatozoa are affected. Inhomogeneous teratozoospermia is frequently associated with oligozoospermia, as discussed above. With regard to homogeneous teratozoospermia, we shall consider macrozoospermia, globozoospermia, acephalic spermatozoa, and multiple morphological abnormalities of the flagella (MMAF) separately. All these syndromes have a frequency below 0.05% and are considered as rare diseases in the Orphanet database (https://www.orpha.net). The genetic transmission of these conditions is always autosomal recessive.

### Macrozoospermia

Macrozoospermia (MIM # 243060, also referred to as macrocephalic sperm head syndrome), was first described in 1977 [22]. The spermatozoa have large, abnormally shaped heads and multiple flagella (usually four). The condition leads to primary infertility, with no chance of paternity; all spermatozoa are aneuploid, and only sperm donation or adoption is possible. This syndrome is due to mutations in the *AURKC* gene [6]. Genetic screening is recommended, with a focus on particular recurrent mutations as a function of the ethnic origin. As WES is only recommended for syndromic men in whom 2 deleterious SNVs have not been identified, its use should be limited to research programs. Lastly, WGS is unlikely to be a first-line test for macrozoospermia in the near future.

### Globozoospermia

Globozoospermia (MIM 613958, a severe form of teratozoospermia with primary infertility) was first described in humans in 1971 [23]. It is characterized by round spermatozoa that lack an acrosome. Hence, the spermatozoa are unable to adhere to and penetrate the zona pellucida. In contrast to macrozoospermia, ICSI gives men with globozoospermia a chance of fatherhood, even if, the absence of phospholipase C zeta prevents oocyte activation after sperm injection [24]. According to the literature, more than 90% of patients with globozoospermia have a *DPY19L2* defect (a homogenous deletion, in 80% of cases). Hence, WES should only be suggested after first-line screening for homozygous *DPY19L2* deletions [25]. WGS is unlikely to become a first-line test in the near future.

A TS strategy could also be proposed in this situation. However, since globozoospermia is rare, there would be no advantage in developing this type of panel for a small number of patients. Prior to clinical validation, this strategy appears to be time-consuming and expensive, considering, at present, the cost of TS similar to that of WES, and the genetic analysis software development. The testing strategy and genetic diagnosis will not modify the clinical management, and IVF (with or without oocyte activation) will be suggested [25]. A genetic diagnosis is only etiological; like all autosomal recessive diseases, globozoospermia does have any consequences for the offspring.

### Acephalic spermatozoa syndrome

Acephalic spermatozoa syndrome (MIM 617187) is a rare condition that was first described in 1979 [26]. The sperm are predominantly headless or lack flagella [27]. In contrast to the syndromes described above, a large variety of genetic abnormalities have been described; hence, it makes sense to use WES (and, when available, WGS) as a first-line test. Similarly, a TS strategy could be suggested, again with the limitations discussed in the previous section. Whatever the strategy or genetic diagnosis, IVF will be the recommended clinical approach.

### Multiple morphological abnormalities of the flagella

Although MMAF is a rare syndrome, cases have been reported regularly since 1984 [28]. Due to peri-axonemal and axonemal defects, the flagella of the sperm in the ejaculate are short, coiled, absent, or of irregular caliber. MMAF is more genetically heterogeneous than the above-mentioned phenotypes, [29], and genetic alterations are consistently reported in literature. Hence, WES appears to be suitable for these patients. Similarly, a TS strategy could be suggested, with the limitations discussed above. Whatever the strategy or genetic diagnosis, the clinical strategy will be IVF.

## Other situations

Male infertility is not limited to sperm defects. In rare cases of idiopathic male infertility, WES might be able to provide a molecular explanation. As new data will be necessary to implement WES for routine diagnosis, this technique is currently limited to use in research programs.

Lastly, the use of WES to explain male infertility is currently subject to debate. It was recently suggested that NGS-based TS of a panel of 110 genes can evaluate spermatogenesis failures, central hypogonadism, androgen insensitivity syndrome, congenital hypopituitarism, and primary ciliary dyskinesia [30]. As expected, this technology clearly increases the diagnosis rate in patients with idiopathic oligozoospermia or NOA and reduces the proportion of idiopathic cases. However, and as explained above, it restricts the identification of variants to previously reported genes, when the cost of TS is quite similar to that of WES, and when more than 2000 genes are considered to be testis-specific. Even though first-line clinical practice will not be modified in the near future, genetic and histological correlations will soon help clinicians to advise infertile men after an initial TESE failure, for example.

In conclusion, WES is already a first- or second-line diagnostic tool. Taking into account the complexity of this practice for the patient, the clinician and the geneticist, WGS will probably replace WES and other molecular genetic analyses in the near future, except in particular situations with a high degree of genetic homogeneity (such as CAVD, macrozoospermia and globozoospermia).

## Data Availability

not applicable.

## Abbreviations

ADGRG2: adhesion g protein-coupled receptor g2
AURKC: aurora kinase C
AZF: azoospermia factor
CBAVD: congenital bilateral absence of the vas deferens
CF: cystic fibrosis
CFTR: cystic fibrosis transmembrane conductance regulator
CNV: copy number variant
DPY19L2: dpy-19-like 2
ICSI: intracytoplasmic sperm injection
IVF: in vitro fertilization
MEI: meiotic double-stranded break formation protein
MMAF: multiple morphological abnormalities of the flagella.
NOA: non-obstructive azoospermia
OA: obstructive azoospermia
PANX2: pannexin 2
SLC9A3: solute carrier family 9, member 3
SNV: single nucleotide variant
STAG: stromal antigen
SYCE1: synaptonemal complex central element protein 1
TESE: testicular sperm extraction
TEX: testis expressed
WES: whole exome sequencing
WGS: whole genome sequencing
